# Palmitic Acid-Enriched Diet Increases α-Synuclein and Tyrosine Hydroxylase Expression Levels in the Mouse Brain

**DOI:** 10.3389/fnins.2018.00552

**Published:** 2018-08-06

**Authors:** Jared Schommer, Gurdeep Marwarha, Kumi Nagamoto-Combs, Othman Ghribi

**Affiliations:** ^1^Department of Biomedical Sciences, School of Medicine and Health Sciences, University of North Dakota, Grand Forks, ND, United States; ^2^Department of Pathology, School of Medicine and Health Sciences, University of North Dakota, Grand Forks, ND, United States

**Keywords:** dopamine, palmitic acid, Parkinson’s disease, α-synuclein, tyrosine hydroxylase

## Abstract

**Background:** Accumulation of the α-synuclein (α-syn) protein and depletion of dopaminergic neurons in the *substantia nigra* are hallmarks of Parkinson’s disease (PD). Currently, α-syn is under scrutiny as a potential pathogenic factor that may contribute to dopaminergic neuronal death in PD. However, there is a significant gap in our knowledge on what causes α-syn to accumulate and dopaminergic neurons to die. It is now strongly suggested that the nature of our dietary intake influences both epigenetic changes and disease-related genes and may thus potentially increase or reduce our risk of developing PD.

**Objective:** In this study, we determined the extent to which a 3 month diet enriched in the saturated free fatty acid palmitate (PA) influences levels of α-syn and tyrosine hydroxylase, the rate limiting enzyme in dopamine synthesis in mice brains.

**Methods:** We fed the m-Thy1-αSyn (m-Thy1) mouse model for PD and its matched control, the B6D2F1/J (B6D2) mouse a PA-enriched diet or a normal diet for 3 months. Levels of α-syn, tyrosine hydroxylase, and the biogenic amines dopamine and dopamine metabolites, serotonin and noradrenaline were determined.

**Results:** We found that the PA-enriched diet induces an increase in α-syn and TH protein and mRNA expression levels in m-Thy1 transgenic mice. We also show that, while it didn’t affect levels of biogenic amine content in the B6D2 mice, the PA-enriched diet significantly reduces dopamine metabolites and increases the level of serotonin in m-Thy1 mice.

**Conclusion:** Altogether, our results demonstrate that a diet rich in the saturated fatty acid palmitate can modulate levels of α-syn, TH, dopamine, and serotonin which all are proteins and neurochemicals that play key roles in increasing or reducing the risk for many neurodegenerative diseases including PD.

## Introduction

Hallmarks of PD include the loss of dopaminergic neurons containing-TH in the *substantia nigrapars compacta* and the abnormal accumulation of α-syn protein in Lewy bodies (Schapira, 1997; Spillantini et al., 1997; Crowther et al., 2000). The role of α-syn in the pathogenesis of PD is not well-understood but extensive experimental data points to a neurotoxic role of high levels of the protein in its soluble and aggregated forms (Snyder and Wolozin, 2004; von Bohlen und Halbach et al., 2004; Adamczyk et al., 2006; Brown, 2010). The causes of PD are likely multi-factorial with genetic predisposition and environmental factors contributing to the pathogenesis of the disease. To date, studies focused on the contributions of dietary fat intake to the risk of PD have yielded inconsistent results (White et al., 2009). Epidemiological studies of dietary fat intake and PD have found either a positive association (Logroscino et al., 1996; Anderson et al., 1999; Johnson et al., 1999; Miyake et al., 2010), no association (Hellenbrand et al., 1996; Tan et al., 2007), or protective effects (Abbott et al., 2003; Chen et al., 2003; de Lau et al., 2005; Powers et al., 2009; Kyrozis et al., 2013; Kamel et al., 2014). Studies focused on specific groups of fatty acids have provided little clarity. Indeed, while poly-unsaturated fatty acids (PUFAs) and mono-unsaturated fatty acids (MUFAs) have been shown to be protective in some stud possible neurodegeneration as thdies (Abbott et al., 2003; de Lau et al., 2005) and detrimental in another (Dong et al., 2014), sFFAs have shown positive associations (Logroscino et al., 1996; Anderson et al., 1999; Johnson et al., 1999; Chen et al., 2003) or no significant relationship with PD risk (Hellenbrand et al., 1996; Chen et al., 2002, 2003; Powers et al., 2003). Additionally, *in vitro* studies have shown that while PUFAs increase α-syn oligomerization and insoluble aggregate formation, sFFAs did not (Sharon et al., 2003; Assayag et al., 2007). Many of these epidemiological studies utilized food frequency questionnaires without clarifying the specific role of each sFFAs. In studies carried out in mice, the n-3 PUFAs eicosapentaenoic acid (EPA) and docosahexaenoic acid (DHA) have been shown to provide neuroprotective effects in animal models of PD (Bousquet et al., 2008, 2011; Seidl et al., 2014; Dyall, 2015). Another study in which m-Thy1 mice were fed a diet enriched in DHA over a 10-month span showed improved survival but no major impact on the dopaminergic system, motor impairments, or brain α-syn levels (Bousquet et al., 2008, 2011; Seidl et al., 2014; Dyall, 2015). Several other animal studies utilized high fat diets that aren’t isocaloric and contain high levels of cholesterol; however these studies didn’t determine the contribution of specific fatty acids (Choi et al., 2005; Bousquet et al., 2012). Therefore, the role of dietary fat in PD risk requires a more precise examination of the contributions of individual fatty acids including saturated fats to elucidate the effects of each fat on PD risk.

The brain contains six saturated fatty acids: myristic acid, palmitic acid, stearic acid, arachidic acid, behenic acid, and lignoceric acid (Julien et al., 2006). We determined the specific effects of a diet rich in palmitic acid, the most abundant sFFA in the body and diet, on key proteins involved in PD risk. PA has been shown to be increased in neurodegenerative disease brains with high levels of this fatty acid found in the frontal cortex of PD (Fabelo et al., 2011) and also in parietal cortex in AD (Fraser et al., 2010). We fed m-Thy1 mice and their controls, the B6D2 mice, an isocaloric diet enriched in PA and examined the effects on the levels of two major hallmarks of PD, α-syn protein and TH, the rate limiting enzyme in dopamine synthesis in the *substantia nigra*. We chose the m-Thy1 mouse model that exhibits many similarities with PD (Rockenstein et al., 2002). The m-Thy1 mouse model overexpresses full-length human wild-type α-synuclein under the murine Thy-1 promoter. They have been extensively characterized and exhibit threefold increase in α-syn protein at 5 months, and decreased TH at 8 months (Rockenstein et al., 2002; Fleming et al., 2004, 2006, 2008; Chesselet et al., 2012; Rabl et al., 2017). Our data shows that the PA diet regulated the expression levels of α-syn, TH, dopamine and serotonin, which are all key proteins and neurochemicals involved in the pathogenesis of neurodegenerative diseases. It is currently unknown if diets rich in other saturated fatty acids such as myristic acid can have the same effects as a PA-enriched diet. Myristic acid has been shown to cause anxiolytic-like effects in rats (Contreras et al., 2014) which suggests it has the ability to alter neurochemical signaling in the brain and also needs to be further explored. Additionally, myristic acid has been suggested to be protective against hyperphosphorylation of the TAU protein that is involved in many neurodegenerative diseases (Ciesielski et al., 2016).

## Materials and Methods

### Feeding Regimens

Mice overexpressing full-length human wild-type α-syn under the murine Thy-1 promoter on the X chromosome were procured from the Chesselet laboratory at the University of California, Los Angeles (UCLA). The corresponding background control B6D2F1/J mice (Stock # 100006) were procured from The Jackson Laboratory (Bar Harbor, ME, United States). We used male animals in these studies because female mice have the ability to inactivate the X chromosome, which may contain the inserted human α-syn gene under the m-Thy1 promoter. All animal procedures were carried out in accordance with the U.S. Public Health Service Policy on the Humane Care and Use of Laboratory Animals and were approved by the Institutional Animal Care and Use Committee at the University of North Dakota (Protocol 1506-2). All animal experiments complied with the National Institutes of Health Guide for the Care and Use of Laboratory Animals (NIH Publications No. 8023, revised 1978). The mice were housed individually in ventilated cages at an ambient room temperature (23–25°C) and ambient relative humidity ranging between 50 and 70%. The mice were maintained on 12:12 h light:dark cycle and allowed access to food and water *ad libitum*. Both genotypes of 3-month-old male mice, m-Thy1 and their backcrossed wild-type B6D2 mice (*n* = 8–9 per group), were fed either a PA-enriched diet (custom-made, TD 1106162, Harlan Teklad, 2.2% w/w palmitic acid) or a control diet (custom made, TD 85172, Harlan Teklad, 0.8% w/w palmitic acid) for 3 months. The diets were isocaloric in relation to each other with the exception of palmitate and linoleate content and based on the NIH-07 open formula. The respective composition of the diets is shown in **Table 1**. Necropsy was performed at six (6) months of age. The genotype of all mice was verified with PCR analysis of tail snip DNA via general endpoint PCR. The *HPRT* gene was used as the internal control with a forward primer of GAAGAGCTACTGTAATGATCAGTCAACGG and a reverse primer of GAGAGGTCCTTTTCACCAGCAAGC. The forward primer used for the human *SNCA* gene was GCTACTGCTGTCACACCCGTC and the reverse primer was GATGATGGCATGCAGCACTGG.

**Table 1:** Composition of the control chow diet and palmitate-enriched diet.

Components	Control chow diet	Palmitic acid-enriched diet
Proteins	23.6% w/w	23.6% w/w
Carbohydrates	65.8% w/w	65.8% w/w
Total fat	5.6% w/w	5.6% w/w
Total energy	4.08 kcal/gram	4.08 kcal/gram
Myristic acid (14:0)	0.1% w/w	0.1% w/w
Palmitic acid (16:0)	0.8% w/w	2.2% w/w
Stearic acid (18:0)	0.2% w/w	0.2% w/w
Palmitoleic acid (16:1)	Trace	Trace
Oleic acid (18:1)	1.2% w/w	1.2% w/w
Gadoleic acid (20:1)	Trace	Trace
Linoleic acid (18:2 n6)	2.2% w/w	0.8% w/w
Linolenic acid (18:3 n3)	0.2% w/w	0.2% w/w
Arachadonic acid (20:4 n6)	Trace	Trace
EPA (20:5 n3)	0.1% w/w	0.1% w/w
DHA (22:6 n3)	0.3% w/w	0.3% w/w

### Western Blotting Analysis

*Substantia nigra*-enriched fractions were prepared as previously described (Marwarha et al., 2010, 2011) and as follows. *Substantia nigra*-enriched tissues (20 mg) were dounce homogenized in RIPA tissue lysis buffer (50 mM Tris, 150 mM NaCl, 0.1% SDS, 0.5% sodium deoxycholate, 1% Triton X, pH 7.4) supplemented with protease and phosphatase inhibitors. The samples were centrifuged at 5000 × *g* for 15 min and the supernatant harvested. Protein concentrations were determined by the Bradford protein assay method. Proteins (10 μg) were resolved on SDS-PAGE gels followed by transfer to a polyvinylidene difluoride (PVDF) membrane (Bio-Rad, Hercules, CA, United States) and incubation with the antibodies listed in **Table 2**. The origin, source, and dilutions of the respective antibodies used for this study are compiled in **Table 2**. β-Actin was used as a gel loading control. The blots were developed with enhanced chemiluminescence (Clarity^TM^ Western ECL blotting substrate, Bio-Rad, Hercules, CA, United States) and imaged using an Aplegen Omega Lum G System (Pleasanton, CA, United States). The analysis was performed using ImageJ (NIH, United States) software. The results were quantified by densitometry and represented as total integrated densitometric values. Data were analyzed using the non-parametric, unpaired Student’s *t*-test with the Mann–Whitney *post hoc* test. Western blots are expressed as fold change over β-Actin (*n* = 4 for B6D2 mice, *n* = 3–4 for m-Thy1 mice) including three technical replicates.

**Table 2:** List of antibodies used in the study.

Antibody	Dilution	Host	Manufacturer	Catalog #	RRID
α-syn	1:500	Rabbit	Cell Signaling	2642S	AB_10695412
TH	1:500	Rabbit	Cell Signaling	2792S	AB_10691683
pS^40^TH	1:500	Rabbit	Sigma Aldrich	T9573	AB_261823

### Real Time-RT PCR

Total RNA was extracted from *substantia nigra*-enriched tissue with the QuickGene RNA cultured cell HC kit S (Autogen, Holliston, MA, United States). Total RNA (0.5 μg) was reverse transcribed into cDNA with qScript cDNA SuperMix (Quanta Biosciences, Gaithersburg, MD, United States). Real-time rtPCR was then performed on the cDNA with taqman probes for the *SNCA* (Mm01188700_m1) and *TH* (Mm00447557_m1) genes (Applied Biosystems, Foster City, CA, United States) and normalized to 18S rRNA. Data were analyzed using the non-parametric, unpaired Student’s *t*-test with the Mann–Whitney *post hoc* test. Real-Time RT-PCR is expressed as fold change over 18S rRNA using the ΔΔC_T_ method (*n* = 4–5) including two technical replicates.

### Immunohistochemistry

The right cerebral hemispheres of m-Thy1 and B6D2 mice were sectioned using a freezing microtome. As previously described (Manocha et al., 2017), multiple paraformaldehyde-fixed and sucrose-equilibrated tissues were embedded in a 15% gelatin (in 0.1 M phosphate buffer, pH 7.4) matrix to form sample blocks for simultaneous processing. The blocks were immersed in a 4% paraformaldehyde solution for 3–4 days to harden the gelatin matrix, followed by a 30% sucrose solution that was replaced every 2 days until the blocks were utilized. The blocks were then flash frozen using dry-ice/isomethylpentane, and 40 μm serial sections were cut using a freezing microtome. Serial sections (960 μm apart) were then immunostained using an anti-TH antibody (1:500 dilution) and an anti α-syn antibody (1:500 dilution, see **Table 2** for detailed descriptions of antibodies). The antigens were visualized using a Vector ABC kit and DAB as the chromogen (Vector Laboratories, Inc., Burlingame, CA, United States) according to the manufacturer’s protocols. The slides were dehydrated through a series of ethanol concentrations and Histo-Clear (National Diagnostics, Atlanta, GA, United States) before being coverslipped using Permount. Photomicrographs were taken using an upright Leica DM1000 microscope and a Leica DF320 digital camera system (*n* = 2).

### Biogenic Amine Analysis Using HPLC-ECD

*Substantia nigra*-enriched tissues were shipped to the Neurochemistry Core at Vanderbilt University where biogenic amine analysis was performed. Briefly, tissue samples were homogenized using a tissue dismembrator in 100–750 ul of 0.1 M TCA, which contains 10^−2^ M sodium acetate, 10^−4^ M EDTA, and 10.5% methanol (pH 3.8). Ten microliters of homogenates were used for the protein assay. The samples were then spun in a microcentrifuge at 10,000 × *g* for 20 min, and the supernatant was removed for biogenic monoamine analysis. Protein concentrations were determined using a BCA Protein Assay Kit (Thermo Scientific). Ten microliters of tissue homogenate was distributed into a 96-well plate, and 200 l of mixed BCA reagent (25 ml of Protein Reagent A mixed with 500 μl of Protein Reagent B) was added. The plate was then incubated at room temperature for 2 h for color development. A BSA standard curve was run at the same time. Absorbance was measured using a plate reader (POLARstar Omega) purchased from BMG LABTECH Company.

Biogenic amine concentrations were determined using an Antec Decade II (oxidation: 0.65) electrochemical detector operated at 33°C. Twenty microliter samples of the supernatant were injected using a Water 2707 autosampler onto a Phenomenex Kintex C18 HPLC column (100 mm × 4.60 mm, 2.6 um). Biogenic amines were eluted with a mobile phase consisting of 89.5% 0.1 M TCA, 10^−2^ M sodium acetate, 10^−4^ M EDTA, and 10.5% methanol (pH 3.8). Solvent was delivered at 0.6 ml/min using a Waters 515 HPLC pump. Using this HPLC solvent, the biogenic amines were eluted in the following order: Noradrenaline, Adrenaline, DOPAC, Dopamine, 5-HIAA, HVA, 5-HT, and 3-MT. HPLC control and data acquisition were managed using Empower software. Isoproterenol (5 ng/mL) was included in the homogenization buffer for use as a standard to quantify the biogenic amines. Data were analyzed using the non-parametric, unpaired Student’s *t*-test with the Mann–Whitney *post hoc* test and are expressed as ng/mg protein (*n* = 3) including three technical replicates.

### Statistical Analysis

Data were analyzed using the non-parametric, unpaired Student’s *t*-test with the Mann–Whitney *post hoc* test. Statistical analysis was performed with GraphPad Prism software 6.07. Western blots are expressed as fold change over β-Actin (*n* = 4) including three technical replicates. Quantitative data from the western blotting analysis are presented as mean ± SEM with unit value assigned to control diet and the extent of differences among the samples being expressed relative to the unit value of control diet. Quantitative data for Real Time-rtPCR analysis are presented as mean ± SEM and expressed as fold-change from control diet. Real-Time RT-PCR for SNCA and TH is expressed as fold change over 18S rRNA using the ΔΔC_T_ method (*n* = 4–5) including two technical replicates.

## Results

### PA-Enriched Diet Exhibit Increased α-Syn Expression Levels

**FIGURE 1:**
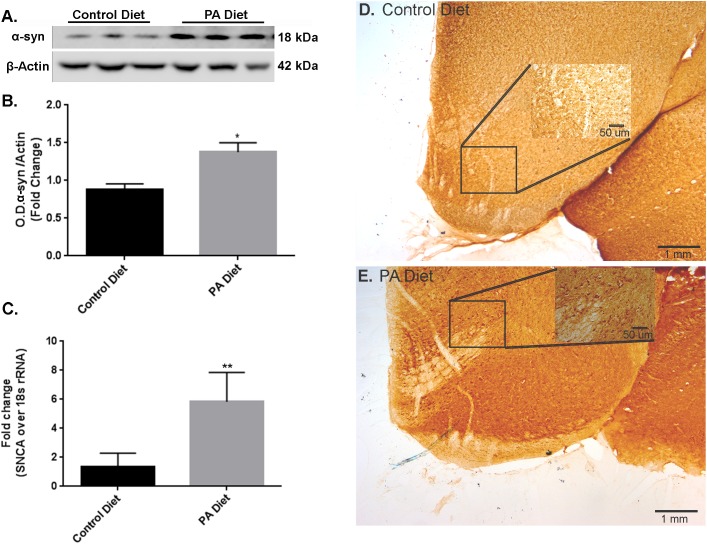
Palmitate (PA)-enriched diet exhibit increases α-syn expression in B6D2 mice. Representative western blot **(A)** and optical density **(B)** of α-syn in the *substantia nigra*-enriched fraction of brains from B6D2 mice showing that the PA diet significantly increases α-syn protein levels. **(C)** Real-time RT-PCR shows that the PA diet increases SNCA mRNA. Immunocytochemistry of the *substantia nigra* shows that the PA diet-fed mice exhibit increased α-syn immunoreactivity **(E)** compared to the control diet **(D)**. ^∗^*p* < 0.05, ^∗∗^*p* < 0.01 versus control diet.

We examined the effects of a PA-enriched diet on α-syn protein levels and mRNA expression in the *substantia nigra*-enriched fractions from the B6D2 mice and found that 3 months of feeding with a PA-enriched diet significantly increased (*p* < 0.05) α-syn protein levels compared to the control diet (**Figures 1A,B**). To determine whether the PA-enriched diet affected α-syn gene expression via transcription, we performed Real-Time RT-PCR and found that the *SNCA* gene was significantly increased (*p* < 0.01) in mice fed PA-enriched chow (**Figure 1C**). We then performed immunohistochemistry and found that the PA-enriched diet that confirms increased positive staining of α-syn (**Figure 1E**) than the control diet (**Figure 1D**). This data suggests that a PA-enriched diet is capable of regulating α-syn at a transcriptional level in B6D2 mice.

**FIGURE 2:**
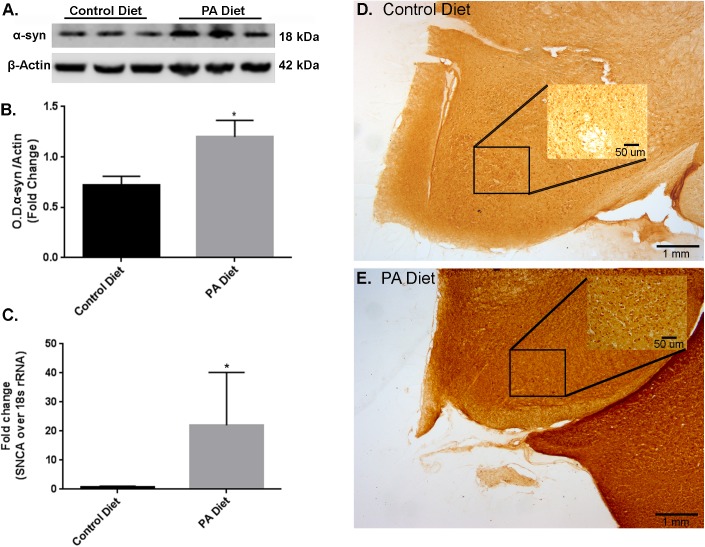
Palmitate-enriched diet increases α-syn expression levels in m-Thy1-mice. Representative western blot **(A)** and optical density **(B)** of α-syn in the *substantia nigra*-enriched fraction of brains from m-Thy1-αsyn mice showing that the PA diet significantly increases α-syn. **(C)** Real-time RT-PCR shows that the PA diet also increases SNCA mRNA. Immunocytochemistry of the *substantia nigra* shows that PA diet-fed mice **(E)** exhibit more immunoreactivity to α-syn antibody than control diet fed mice **(D)**. ^∗^*p* < 0.05 versus control diet.

We also examined the effects of a PA-enriched diet on α-syn protein and mRNA expression levels in the *substantia nigra*-enriched fractions from m-Thy1 mice and found that 3 months of a PA-enriched diet feeding significantly increased (*p* < 0.05) α-syn protein levels as demonstrated by western blotting (**Figures 2A,B**). To determine whether the PA-enriched diet also affects α-syn gene expression, we performed Real-Time RT-PCR and found that the *SNCA* gene was significantly increased (*p* < 0.05) in mice fed PA-enriched diet (**Figure 2C**). The RT-PCR primers chosen were used to assess the effects of a PA-enriched diet on endogenous expression of mouse SNCA. Human primers were also used for the samples which showed no amplification in the aforementioned B6D2 mice while the m-Thy1 mice showed no significant amplification differences suggesting that the PA-enriched diet does not affect the m-Thy1 promotor upon which the inserted human SNCA is located (data not shown). This data suggests the elevated levels of α-syn are due to the induction of increased endogenous mouse α-syn. We then performed immunohistochemistry and found that the PA-enriched diet resulted in increased α-syn immunostaining (**Figure 2E**) compared to the control diet-fed mice (**Figure 2D**). The cellular localization of the protein in both strains of animals on both diets seems to be in the soma as we were unable to identify any significant differences in distribution in the nucleus. We did not observe lewy-body-like inclusions in addition to the increase in α-syn protein levels. A longer duration of the diet may lead to the formation of lewy-body-like inclusions and possible neurodegeneration as the diet seems to be causing detrimental effects in regard to increased α-syn but also elicits protective effects that will be elaborated upon in the forthcoming data. This data suggests that a PA-enriched diet is capable of regulating α-syn expression levels in m-Thy1 mice.

### PA-Enriched Diet Increases TH Expression Levels

**FIGURE 3:**
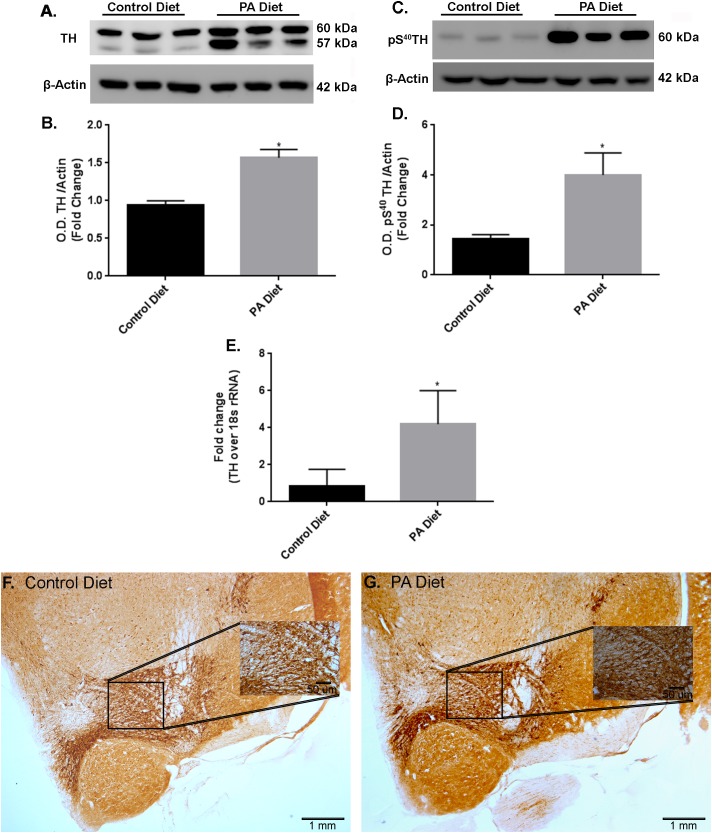
B6D2 mice on a PA diet exhibit increased TH and pS^40^TH expression. Representative western blot **(A,C)** and optical density **(B,D)** showing the PA diet significantly increases levels of TH and pS^40^TH respectively in the *substantia nigra*-enriched fraction of brains from B6D2 mice. Real-time RT-PCR shows that the PA diet significantly increases TH mRNA **(E)**. Immunocytochemistry shows that PA diet-fed mice exhibit increased immunoreactivity to TH antibody **(F)** compared to control diet-fed **(G)**. ^∗^*p* < 0.05 versus control diet.

We examined the effects of a PA-enriched diet on TH protein and mRNA expression levels in the *substantia nigra*-enriched fractions of brains from B6D2 mice and found that 3 months of feeding with a PA-enriched diet significantly increased (*p* < 0.05) TH protein levels compared to the control diet (**Figures 3A,B**). In addition, levels of phospho S^40^TH, the active form of TH (Dunkley et al., 2004) were also shown to be significantly increased (*p* < 0.05) following PA-enriched diet feeding (**Figures 3C,D**). To assess whether the PA-enriched diet affects *TH* gene expression via transcription, we performed Real-Time RT-PCR and found that the *TH* gene expression was significantly increased (*p* < 0.05) in mice fed the PA-enriched diet compared to control diet (**Figure 3E**). Immunohistochemistry shows that the PA-enriched diet increases TH staining (**Figure 3G**) compared with the control diet-fed mice (**Figure 3F**). This data suggests that in B6D2 mice, a PA-enriched diet is capable of regulating TH at a transcriptional and translational level.

**FIGURE 4:**
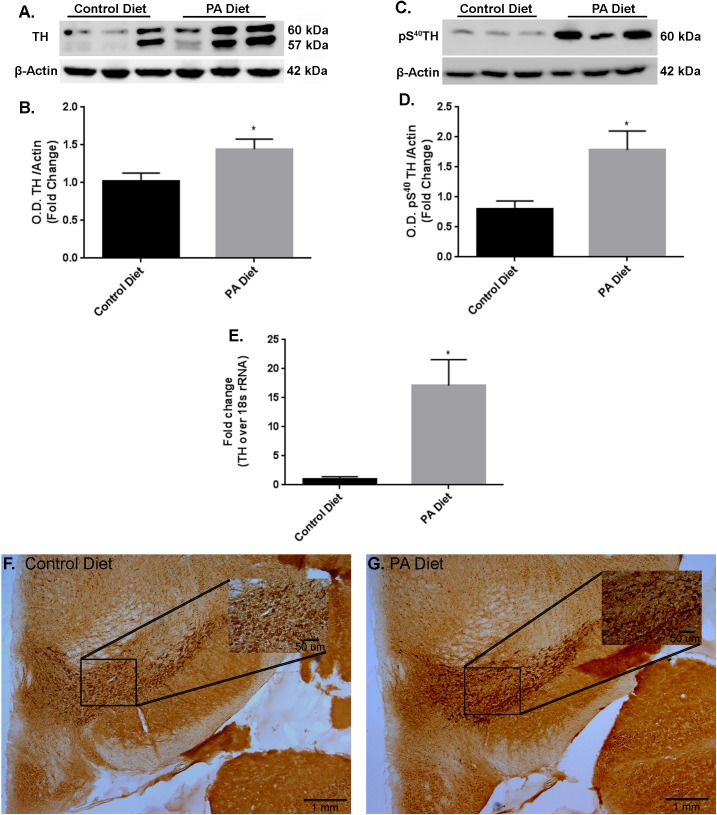
m-Thy1-αSyn mice on a PA diet exhibit increased TH and pS^40^TH expression. Representative western blot **(A,C)** and optical density **(B,D)** showing the PA diet significantly increases levels of TH and pS^40^TH respectively in the *substantia nigra*-enriched fraction of brains from m-Thy1-αSyn mice. Real-time RT-PCR shows that the PA diet significantly increases TH mRNA **(E)**. Immunocytochemistry shows that PA diet-fed mice exhibit increased immunoreactivity to TH antibody **(G)** compared to control diet **(F)**. ^∗^*p* < 0.05 versus control diet.

In the *substantia nigra*-enriched fraction of m-Thy1 mice, the 3 months of feeding with a PA-enriched diet significantly increases (*p* < 0.05) TH protein levels (**Figures 4A,B**). In addition, levels of pS^40^TH were also shown to be significantly increased (*p* < 0.05) with the PA-enriched diet (**Figures 4C,D**). Real-Time RT-PCR shows that the *TH* gene was significantly increased (*p* < 0.05) in mice fed the PA-enriched chow (**Figure 4E**). We then performed immunohistochemistry in the *substantia nigra* and found that the PA-enriched diet resulted in increased TH staining (**Figure 4G**) compared with the control diet-fed mice (**Figure 4F**). This data suggests that in m-Thy1 mice a PA-enriched diet can regulate TH at a transcriptional and translational level. Not only did the PA diet increase TH but it also increased pS^40^TH in both B6D2 and m-Thy1 mouse models.

### PA Diet Differently Affects Biogenic Amines

**FIGURE 5:**
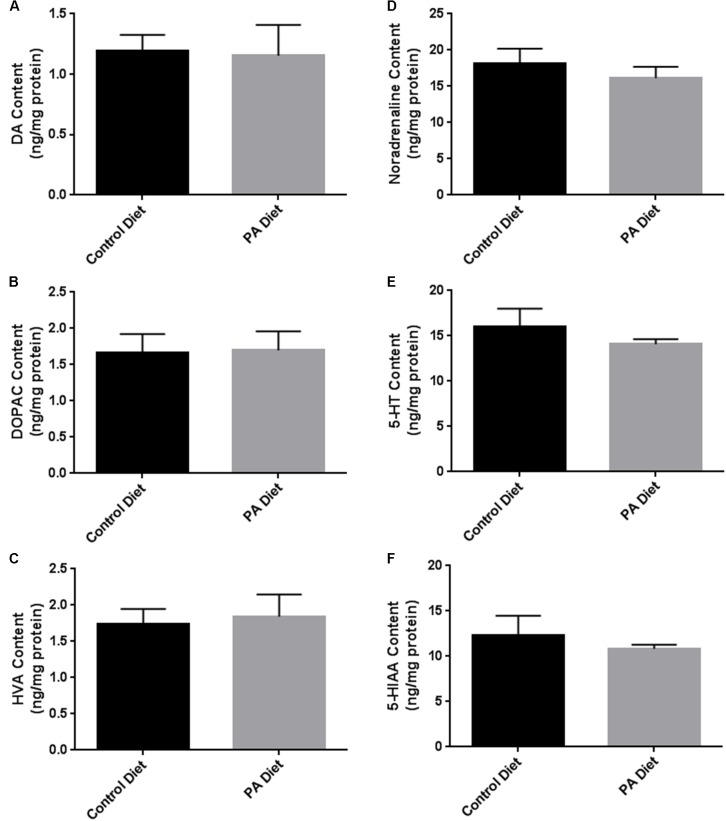
Biogenic amine analysis revealed no significant differences in biogenic amines between B6D2 mice on a control or PA diet. PA-enriched diet doesn’t affect DA **(A)**, DOPAC **(B)**, HVA **(C)**, Noradrenaline **(D)**, Serotonin (5-HT) **(E)**, or 5-HIAA **(F)** content compared to control diet. Data is expressed as ng/mg protein.

**FIGURE 6:**
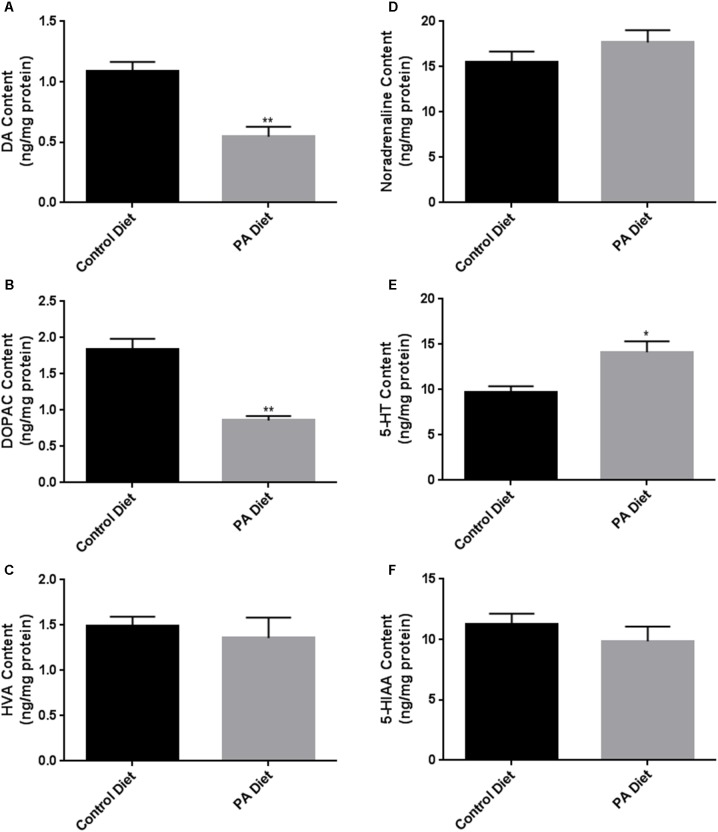
Biogenic amine analysis shows significant differences in biogenic amine levels between control and PA diet-fed m-Thy1 mice. While PA-enriched diet reduced DA **(A)** and DOPAC **(B)**, it increased 5-HT content **(E)** and didn’t significantly affect Noradrenaline **(D)**, HVA **(C)**, or 5-HIAA **(F)** levels. Data were analyzed using the non-parametric, unpaired Student’s *t*-test with the Mann–Whitney *post hoc* test. Data is expressed as ng/mg protein. ^∗^*p* < 0.05, ^∗∗^*p* < 0.01 versus control diet.

We assessed the levels of various biogenic amines in the *substantia nigra*-enriched fraction of B6D2 mice fed the control or the PA diets and observed no significant differences in dopamine (DA) content (**Figure 5A**) or its metabolites DOPAC (**Figure 5B**) and HVA (**Figure 5C**) between the two feeding regimens. Also, no significant differences in noradrenaline (**Figure 5D**), serotonin (**Figure 5E**), and the serotonin metabolite 5-HIAA (**Figure 5F**) were observed. Although the PA-enriched diet increased the expression of α-syn and TH, this data suggests the diet does not alter normal biogenic amine content in B6D2 mice. In the *substantia nigra*-enriched fractions of m-Thy1 mice we observed reduced levels of DA (**Figure 6A**) and DOPAC (**Figure 6B**), and increased levels of serotonin (**Figure 6E**) in mice fed a PA-enriched diet, while HVA (**Figure 6C**), Noradrenaline (**Figure 6D**), and 5-HIAA (**Figure 6F**) levels were unchanged between PA-enriched and control diets.

## Discussion

In this study, we determined the specific contribution of the fatty acid PA in regulating expression levels of α-syn and TH, two proteins that are tightly linked to PD, in the m-Thy1 mouse model of PD and its matched control the B6D2 mouse. We found that the PA-enriched diet increased α-syn protein levels and mRNA content in both strains of mice. We also found that in both strains of mice the PA-enriched diet increases the protein and mRNA levels of TH, the rate limiting enzyme in the synthesis of dopamine (Sjoerdsma et al., 1965). Our results suggest that the PA diet may have both protective and destructive effects by increasing TH and α-syn expression levels respectively.

Palmitate (16:0) is the most abundant sFFA acid in the body and the most abundant sFFA in certain foods including meats, cheeses, and dairy products. It is also synthesized *de novo* in the body and makes up 24% of total fatty acids in our blood and 28% of total fatty acids in our CSF (Guest et al., 2013). Numerous *in vitro* studies have focused on various roles of palmitic acid. It has been shown to increase ER stress (Marwarha et al., 2016), proinflammatory cytokine expression in astrocytes and microglia (Gupta et al., 2012; Tracy et al., 2013), activation of TLRs via NFKβ (Oberbach et al., 2012), and to reduce the expression of insulin-degrading enzyme (IDE), a protease responsible for the degradation of amyloid-β, the accumulation of which is implicated in the pathogenesis of AD (Du et al., 2010). However, a role of PA in pathological hallmark formation of PD-type synucleinopathy remain unknown. Human research studies of sFFA have found positive associations (Logroscino et al., 1996; Anderson et al., 1999; Johnson et al., 1999; Chen et al., 2003) or no significant relationship (Hellenbrand et al., 1996; Chen et al., 2002, 2003; Powers et al., 2003) with PD risk. While these studies provide important information, they utilized food frequency questionnaires in which individuals described what they have consumed. This type of survey can be difficult to interpret because individuals may not accurately report what, when, and how much they consumed.

Abnormal accumulation of α-syn protein is a characteristic of PD and other synucleinopathies. While the cause of the accumulation remains unknown, genetic predisposition along with environmental factors are likely to contribute to the pathogenesis of PD. *In vitro* studies have shown that PUFAs increase α-syn oligomerization and insoluble aggregate formation while sFFAs do not (Sharon et al., 2003; Assayag et al., 2007). Additionally, α-syn has been proposed to act as a lipid carrier to shuttle fatty acids around the cell (George and Yang, 2013) and a previous study showed that when α-syn is ablated in primary astrocytes, PA incorporation into membranes is decreased (Castagnet et al., 2005). The increase in PA content in our study may therefore lead to increased α-syn expression, which could function to properly traffic the excess PA to lipid membranes or to the mitochondria for β-oxidation and might therefore be functioning as a protective measure to maintain normal lipid homeostasis.

Tyrosine hydroxylase is a very important enzyme in the synthesis of dopamine (Sjoerdsma et al., 1965) and has been shown to decrease in PD (Tabrez et al., 2012; Zhu et al., 2012). Short chained fatty acids have been shown to upregulate TH expression through a cAMP dependent mechanism (Mally et al., 2004; DeCastro et al., 2005) while the role of long chain saturated and unsaturated fatty acids remains to be determined. In this study, we found that a PA-enriched diet increases TH protein and mRNA expression in both strains of mice. cAMP response element binding protein (CREB) has been shown to be the mediator by which cAMP upregulates TH expression in PC12 cells (Piech-Dumas and Tank, 1999). It is possible that longer chain fatty acids like palmitic acid may have the same effect on CREB activation and are yet to be determined in future studies. To the best of our knowledge, we are the first to show that a PA enriched diet can induce TH protein and mRNA expression. Elucidation of the mechanism by which PA enriched diet induces TH expression is of great importance in the search for disease altering therapies in PD.

We did not observe any significant difference in the levels of biogenic amines in the control B6D2 mice fed the PA-enriched diet compared to the control diet. However, dopamine and serotonin levels were significantly modulated by the PA-enriched diet in the m-Thy1 mice. Our findings demonstrating reduced dopamine content in m-Thy1 mice are intriguing as we show that the PA diet increases TH expression. The contrasting up-regulation of active pS^40^TH vs. reduction of dopamine levels in m-Thy1 mice is curious but may be potentially explained by the observation that a PA-enriched diet exhibits protective and detrimental effects at the same time. The m-Thy1 mice already have an overabundance of α-syn protein levels and the diet exacerbates this effect which may be leading to neuroinflammation and some dopaminergic neuronal death and hence a reduction in global dopamine. On the other hand, the PA-enriched diet clearly upregulates TH protein and mRNA expression and pS^40^TH protein levels in both strains of mice which may be acting as a protective effect. TH catalyzes the conversion of the amino acid L-tyrosine to L-3,4-dihydroxyphenylalanine (L-DOPA) (George and Yang, 2013). It does so by using molecular oxygen (O_2_), in addition to iron (Fe^2+^) and tetrahydrobiopterin as cofactors. Although pS^40^TH is increased in m-Thy1 mice, cofactors may not be present in the m-Thy1 mice leading to less dopamine production. TH is subject to feedback inhibition by all the catecholamines by competing for the binding site of TH with the pterin cofactor (Zigmond et al., 1989; Daubner et al., 2011). Additionally, the observed significant increase in serotonin levels may be inhibiting the function of TH and pS40TH leading to the observed decrease in dopamine levels. Our speculation is supported by previous studies showing that serotonin can reduce the activity of TH. Indeed, serotonin treatment of undifferentiated human neuroblastoma LAN-5 cells led to a decrease in TH activity (John et al., 1991). As such, our findings that PA increases serotonin levels are of significance as an increase is desirable in many conditions such as depression that is frequently associated with PD presentation. The significant increase in serotonin could be explained by the fact that sFFAs have been shown to upregulate the serotonin receptor Htr2c and downregulate monoamine oxidase enzymes (Cataldo et al., 2016). The PA-enriched diet may thus result in more serotonin availability.

## Conclusion

In summary, we demonstrate that the PA-enriched diet induces an increase in α-syn and TH protein and mRNA expression in both B6D2 and m-Thy1 mice. To the best of our knowledge, our study is the first to show that a diet enriched in PA increases the levels of TH protein and mRNA in these mouse models. We also show that the PA-enriched diet does not affect biogenic amine content in control B6D2 mice but significantly changes dopamine and serotonin levels in m-Thy1 mice relative to control-fed mice. Altogether, our results demonstrate that a diet enriched in PA increases the levels of TH, and serotonin, an effect that can provide beneficial effects in a variety of conditions. Future studies are needed to elucidate the mechanisms by which a PA-enriched diet modulates these proteins.

## Availability of Data and Material

The datasets used and/or analyzed during the current study are available from the corresponding author on reasonable request.

## Author Contributions

JS wrote the manuscript, designed experiments, acquired, analyzed, and interpreted data. GM contributed to the conception of experiments, interpretation, and critically revised the manuscript. KN-C aided and trained others on technical aspects. OG conceived the idea, designed experiments, revised the manuscript, and gave final approval of the version to be published. All authors read and approved the final manuscript.

## Conflict of Interest Statement

The authors declare that the research was conducted in the absence of any commercial or financial relationships that could be construed as a potential conflict of interest.

## Abbreviations

m-Thy1: m-Thy1-αSyn mouse model
α-syn: α-synuclein
PA: palmitic acid
PA diet: palmitic acid-enriched diet
PD: Parkinson’s disease
sFFA: saturated free fatty acids
TH: tyrosine hydroxylase.
